# Validation of plasma protein glycation and oxidation biomarkers for the diagnosis of autism

**DOI:** 10.1038/s41380-023-02357-9

**Published:** 2023-12-22

**Authors:** Aisha Nasser J. M. Al-Saei, Wared Nour-Eldine, Kashif Rajpoot, Noman Arshad, Abeer R. Al-Shammari, Madeeha Kamal, Ammira Al-Shabeeb Akil, Khalid A. Fakhro, Paul J. Thornalley, Naila Rabbani

**Affiliations:** 1 College of Medicine, QU Health, Qatar University, PO Box 2713 Doha, Qatar; 2 Qatar Biomedical Research Institute, Hamad Bin Khalifa University, Qatar Foundation, PO Box 34110 Doha, Qatar; 3 University of Birmingham Dubai, Dubai International Academic City, PO Box 341799 Dubai, UAE; 4 BIOMISA Laboratory, Department of Computer & Software Engineering, National University of Science & Technology (NUST), Islamabad, Pakistan; 5 Department of Pediatrics, Sidra Medicine, P.O. Box 26999 Doha, Qatar; 6 Department of Genetic Medicine, Weill Cornell Medical College, Doha, P.O. Box 24144 Doha, Qatar; 7 Precision Medicine in Diabetes Prevention Laboratory, Population Genetics, Sidra Medicine, P.O. Box 26999 Doha, Qatar; 8 Laboratory of Genomic Medicine-Precision Medicine Program, Sidra Medicine, P.O. Box 26999 Doha, Qatar; 9 College of Health and Life Sciences, Hamad Bin Khalifa University, Doha, P.O. Box 34110 Doha, Qatar

**Keywords:** Diagnostic markers, Biochemistry, Autism spectrum disorders

## Abstract

Autism Spectrum Disorder (ASD) is a common neurodevelopmental disorder in children. It is currently diagnosed by behaviour-based assessments made by observation and interview. In 2018 we reported a discovery study of a blood biomarker diagnostic test for ASD based on a combination of four plasma protein glycation and oxidation adducts. The test had 88% accuracy in children 5–12 years old. Herein, we present an international multicenter clinical validation study (*N* = 478) with application of similar biomarkers to a wider age range of 1.5–12 years old children. Three hundred and eleven children with ASD (247 male, 64 female; age 5.2 ± 3.0 years) and 167 children with typical development (94 male, 73 female; 4.9 ± 2.4 years) were recruited for this study at Sidra Medicine and Hamad Medical Corporation hospitals, Qatar, and Hospital Regional Universitario de Málaga, Spain. For subjects 5–12 years old, the diagnostic algorithm with features, advanced glycation endproducts (AGEs)—N^ε^-carboxymethyl-lysine (CML), N^ω^-carboxymethylarginine (CMA) and 3-deoxyglucosone-derived hydroimidazolone (3DG-H), and oxidative damage marker, *o*,*o*’-dityrosine (DT), age and gender had accuracy 83% (CI 79 – 89%), sensitivity 94% (CI 90–98%), specificity 67% (CI 57–76%) and area-under-the-curve of receiver operating characteristic plot (AUROC) 0.87 (CI 0.84–0.90). Inclusion of additional plasma protein glycation and oxidation adducts increased the specificity to 74%. An algorithm with 12 plasma protein glycation and oxidation adduct features was optimum for children of 1.5–12 years old: accuracy 74% (CI 70–79%), sensitivity 75% (CI 63–87%), specificity 74% (CI 58–90%) and AUROC 0.79 (CI 0.74–0.84). We conclude that ASD diagnosis may be supported using an algorithm with features of plasma protein CML, CMA, 3DG-H and DT in 5–12 years-old children, and an algorithm with additional features applicable for ASD screening in younger children. ASD severity, as assessed by ADOS-2 score, correlated positively with plasma protein glycation adducts derived from methylglyoxal, hydroimidazolone MG-H1 and N^ε^(1-carboxyethyl)lysine (CEL). The successful validation herein may indicate that the algorithm modifiable features are mechanistic risk markers linking ASD to increased lipid peroxidation, neuronal plasticity and proteotoxic stress.

## Introduction

Autism Spectrum Disorders (ASD) is a prenatal disorder which originates in the first trimester of pregnancy and affects 78 million people worldwide [1, 2]. It has high heritability [3], which may reflect genetic vulnerability to shared environmental exposures [4]. Major concerns for subjects with suspected ASD, their parents, and carers are timely access to clinical diagnosis. Guidelines for diagnosis of ASD recommend involvement of a multidisciplinary team of child and adolescent psychiatrists, child neurologists, developmental-behavioural paediatricians, or child psychologists. ASD diagnosis is based on assessments in structured observations, interviews and examinations, medical/developmental review, and assessment instruments. It is currently standardized to the Diagnostic and Statistical Manual of Mental Disorders-5 criteria (DSM-5) with recommended duration of the diagnostic procedure of 3–6 months [5]. Due to a global shortage of specialists trained to assess suspected children using these established criteria, and the growing prevalence of the condition, diagnosis is often preceded by a long delay, in some cases greater than one year, from first referral to expert team evaluation [2].

There is an unmet clinical need for diagnostic techniques based on biomarkers which corroborate well with diagnosis of ASD by experts in child development [2]. The consensus report by the American Psychiatric Association (APA) Work Group on Neuroimaging Markers of Psychiatric Disorders proposed that a promising biomarker-based test for diagnosis of ASD should meet threshold classification criteria of at least 80% specificity and sensitivity [6]. A recent systematic review found no biomarker for diagnosis of ASD meeting these criteria with evidence from two or more independent studies in agreement [7].

A diagnostic aid combining behavioural features from caregiver and healthcare provider questionnaires and home videos recently received regulatory approval by USA Food and Drug Administration (FDA) with sensitivity 98% and specificity 79% but had a high no response rate, 68% [8]. Other studies using magnetic resonance imaging (MRI) [9], visual attention/eye movement or eye tracking assessments [10, 11], genetic mutation assessments [12] and post-natal blood tests for ASD based on transcriptomic analysis of peripheral blood mononuclear cells and proteomics and metabolomics analysis of plasma have been reported but require validation [13–16].

In 2018, we reported a discovery study of a blood test which met the APA Work Group threshold classification criteria. It was based on an algorithm with features of plasma protein glycation and oxidation adducts. A combination of four plasma protein glycation and oxidation adducts—namely *N*^ε^-carboxymethyl-lysine (CML), *N*^ω^-carboxymethyl-arginine (CMA), 3-deoxyglucosone-derived hydroimidazolone (3DG-H) and dityrosine (DT)—gave a diagnostic algorithm with accuracy 88%, sensitivity 92% and specificity 84% in children 5–12 years old [17]. Plasma protein glycation and oxidation adducts occur mostly in albumin, accounting for 60% of plasma protein, which has a half-life of 3 weeks [18]. Plasma protein glycation and oxidation adduct levels thereby reflect changes in precursor glycation and oxidation processes occurring over the 3–4 weeks preceeding blood sampling [19]. Albumin in cerebrospinal fluid (CSF) exchanges relatively rapidly with albumin in plasma (half-life 3.3 h [20]), so albumin modifications detected in plasma have contributions from those occurring in CSF.

Herein, we describe an international multicenter clinical validation study of the plasma protein glycation and oxidation biomarker blood test for ASD. We were able to successfully validate the 4-feature algorithm for classification of children with ASD or typical development (TD) with a similar accuracy to the discovery study in children 5 – 12 years old.

## Materials and methods

### Subject recruitment

**Fig. 1:**
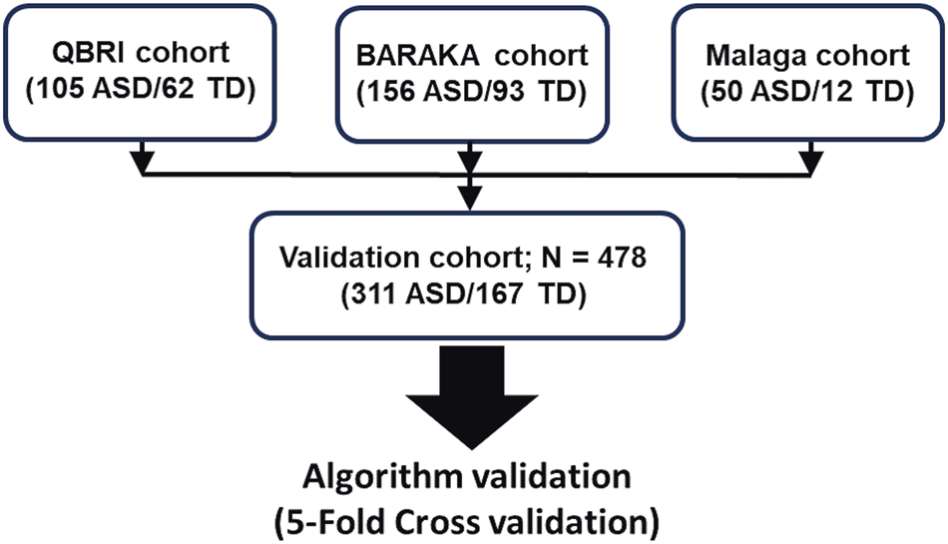
Subjects recruited in the "Blood test for autism" validation study. Subject recruitment by cohort: QBRI, BARAKA, and Malaga cohorts. ASD children with autism spectrum disorder, TD children with typical development.

A total of 478 children were recruited for this study: 311 had a diagnosis of ASD (247 males and 64 females) and 167 were classified as TD children (94 males and 73 females). They were recruited in three cohorts. Firstly, Qatar Biomedical Research Institute (QBRI) cohort (*n* = 167)—recruited at the Child Development Centre, Rumailah Hospital, Hamad Medical Corporation (HMC), Doha, Qatar (subjects with ASD) and Al-Wajbah Health Centre, Primary Health Care Corporation (PHCC), Doha, Qatar (TD children); Project lead Dr Abeer R. Al-Shammari. Secondly, the BARAKA cohort—recruited at Sidra Medicine, Doha, Qatar (*n* = 249). Plasma samples collected from unaffected recruited siblings served as the control population; project lead Dr Kalid Fakhro. Thirdly, Malaga cohort (*n* = 62)—recruited at Hospital Regional Universitario de Málaga, Málaga, Spain; Project lead Dr Yolanda de Diego-Otero (Fig. 1). For QBRI and BARAKA cohorts, children with ASD received a diagnosis of ASD by two child development experts, according to the DSM-5 criteria [5]. For the Malaga cohort, children with ASD were initially identified by completion of the Q-CHAT10 questionnaire [21] completed by parents and paediatrician. ASD diagnosis was further confirmed by ADI-R evaluation [22] by a trained psychiatrist at the Department of Mental Health, Regional University Hospital of Malaga, Spain. ASD severity assessment by Autism Diagnostic Observation Schedule-2 (ADOS-2) [23] was recorded for the QBRI cohort. Children 1.5–12 years of age with ASD or with TD were recruited for this study. For both ASD and TD subjects, inclusion criteria were: no family history of ASD; no immune conditions, such as autoimmune disease, asthma, allergy, and eczema; no neurological conditions, such as epilepsy; no suspected vision, hearing or walking problems; no other health problems, such as cardiovascular, lung, and kidney diseases; and taking any medications and did not have any recent infection or vaccination at the time of study enrolment. Exclusion criteria were: any surgery intervention in the four months prior to blood sample donation. Comorbidities were: attention deficit hyperactivity disorder (ADHD), epilepsy and anxiety. Children with TD were recruited in the local community, with no sign of cognitive, learning, and psychiatric involvement. They were attending mainstream school and had not been subjected to stressful events. TD subjects in the QBRI cohort were also screened using the Social Communication Questionnaire (lifetime version) with a cutoff score <12 for eligibility to exclude the risk of ASD.

The study was reviewed and approved by the Institutional Review Board (IRB), Qatar University (approval numbers: QU-IRB 1599-E/21 and 2019-003). QBRI cohort study was reviewed and approved by the IRB of HMC (approval number: MRC-02-18-116; ASD subjects) and IRB of PHCC (approval number: 2020/06/064; TD subjects). Baraka cohort study received ethical approval of the IRB at Sidra Medicine (approval number: 1500767) and HMC (approval number: MRC-03-20-515). Malaga cohort study received ethical approval by the Ethical Committee of the Regional University Hospital of Malaga, University of Malaga, Malaga, Spain. The experiments conformed to the principles set out in the World Medical Association Declaration of Helsinki. Whole blood samples were collected from children with written informed consent of a parent of all eligible children prior to enrolment, data, and sample collection.

### Blood sampling

Blood samples collected from children with ASD or TD were drawn and processed under the same conditions except for the Malaga cohort blood samples were drawn after 8 h fasting whereas the others were not. Blood donations were processed in the research laboratory within two hours of sample collection. There was no site-specific difference in plasma protein modification contents; *cf*. assays of albumin glycated by glucose, glycated albumin, where fasting and non-fasting sampling gives similar estimates [24]. Ethylenediaminetetra-acetic acid (EDTA) was used as anticoagulant. Plasma and blood cells were separated immediately by centrifugation and stored at –80 °C until analysis and transferred between collaborating laboratories and recruitment sites on dry ice.

### Assay of markers of plasma protein glycation, oxidation and nitration

The content of glycated, oxidized and nitrated adduct residues in plasma protein was quantified in exhaustive enzymatic digests of washed plasma protein extracts by stable isotopic dilution analysis liquid chromatography-tandem mass spectrometry (LC-MS/MS), with correction for autohydrolysis of hydrolytic enzymes, as described previously [17] except a similar updated model of tandem mass spectrometer, Xevo-TQXS (Waters, Manchester, U.K.), was used. Analytes determined were: glycation adducts—*N*^ε^-fructosyl-lysine (FL) and advanced glycation endproducts (AGEs)—CML, *N*^ε^(1-carboxyethyl)lysine (CEL), CMA, glyoxal-derived hydroimidazolone (G-H1), methylglyoxal-derived hydroimidazolone (MG-H1), 3DG-H and glucosepane (GSP); oxidation adducts—methionine sulfoxide (MetSO), DT, N’-formyl-kynurenine (NFK), and nitration adduct, 3-nitrotyrosine (3-NT); and related amino acids precursors, arginine, lysine, methionine, tyrosine and tryptophan. Oxidation, nitration and glycation adduct residues are normalised to their amino acid residue precursors and given as mmol/mol amino acid modified. Average (AVE) additional protein glycation and oxidation adduct variable was the mean value of all 12 plasma protein modifications measured. Sample classification was hidden from the investigators performing sample analysis and data processing.

### Machine learning analysis

The objective was to distinguish between children with ASD or TD. The initial objective was to validate a classifier algorithm with features of plasma protein CML, CMA, DT and 3DG-H in subjects of 5–12 years old, as developed in the discovery study [17]. We also explored if classification performance could be further improved by addition of subject age, gender and additional plasma protein glycation and oxidation adduct features. A secondary objective was to assess the classification performance of the algorithm over a wider age range, including children from 1.5 years old. In all data analysis, the diagnostic algorithms were trained on 80% subjects (training subset) before being used to predict the ASD or TD class for each sample in the remaining subjects (test set) through a fivefold cross-validation process. The outcome was to assign, for each test set sample, a set of probabilities corresponding to each of the ASD/TD groups—the group assignment being that for which the probability is highest. Test data were held separate from algorithm training; algorithm settings were not adjusted once we began to analyse the test set data—thereby guarding against overfitting and hence providing a rigorous estimate of predictive performance. Support Vector Machines (SVMs) algorithms had previously proven optimum [17] whereas herein ensemble classifier gave the best outcome. In addition to algorithm methods tried previously, we also used extreme gradient boosting (XG boost) [25]. In the larger validation set of more diverse age and gender proportions, subject age and gender also emerged as features improving the classification. For algorithm training and testing in the cohort with subjects <5 years old, algorithm training involving the complete panel of protein glycation and oxidation as features was employed. The aim during the training was to select the set of features that accomplishes the highest performance – as judged by classification accuracy. For each performance metric, the mean and 95% CI was determined and reported. The algorithm training and testing was repeated 5 times, following the 5-fold cross-validation process, without altering the algorithm parameters, with 80:20% data split, to test for algorithm’s robustness against any bias towards data split. We developed our computer programs using Statistics, Machine Learning Toolbox of MATLAB^®^ (MathWorks, Inc., Natick, USA) and Python with open-source libraries: Sci-kit learn, Scipy and AutoGluon.

### Statistical analysis

Data are presented as mean ± SD for parametric distributions and median (lower—upper quartile) for non-parametric distributions. The test for normality of data distribution applied was the Kolmogorov–Smirnov test. 95% Confidence intervals (CI) are given for classification performance variables. Significance was evaluated by Student’s *t* test or by Mann–Whitney U-test for parametrically or non-parametrically distributed data, respectively. Bonferroni correction was made for analysis of multiple analytes without preconceived hypothesis. Correlation analysis was performed by the Spearman’s rho method with continuous variables. For ADOS-2 categorical variables with ≥6 categories recorded, Spearman correlation was performed—assuming approximation to a continuous variable [26]. Data were analysed using SPSS, version 24.0.

For power analysis to deduce the number of subjects required for the study design, we based power calculations on variance of area-under-the-curve of receiver operating characteristic plot (AUROC) [27]. Assuming a normal distribution, α confidence level and precision of sensitivity and specificity of 0.05, we designed the study for validation of the autism blood test to classify subjects 5–12 year old with an AUROC variance of 0.06. Power calculations indicate a mean of 92 subjects for case and control study groups is required. This was met by subject recruitment for this study; mean case and control subjects recruited was 92.5. For application of the autism blood test to subjects 1.5–<5 years old, we allowed for a greater data dispersion with AUROC variance of 0.09. Power calculations indicate a mean of 138 subjects for case and control study groups is required. This was slightly exceeded by recruitment in our study where the mean of case and control subjects recruited was 146.5. This allows for up to *ca*. 5% outliers and extreme values.

## Results

### Cohort characteristics

Clinical characteristics of children recruited for the study are given in Table 1. Subject age was not significantly different between children with ASD (5.2 ± 3.0 years) and children with TD (4.9 ± 2.4 years). The ratio of male to female was 1.3 in children with TD and 3.9 in children with ASD. Most study participants (416 of 478) were recruited in Qatar. The recent estimate of prevalence of ASD in Qatar was 1.14% in 6 to 11-year-old children [28]. Sixty-eight percent of children with ASD had mild-to-moderate symptoms and the remaining 32% had severe symptoms. There was minor presence of comorbidities of which ADHD was highest at 5%.

**Table 1:** Cohort demographic and clinical features.

Variable	TD	ASD
All subjects		
N	167	311
Gender (M/F)	94/73	247/64
Age (years)	4.9 ± 2.4	5.2 ± 3.0
Severity of ASD (mild-to-moderate/severe)	—	210/101
Comorbidities (ADHD/epilepsy/anxiety)	—	17/3/1
Subjects >60 months old		
N	69	116
Gender (M/F)	28/41	98/18
Age (years)	8.1 ± 2.3	7.6 ± 2.0
Subjects <60 months old		
N	98	195
Gender (M/F)	66/32	149/46
Age (years)	3.1 ± 0.9	3.1 ± 0.7

### Plasma protein glycation and oxidation

Plasma protein content of glycation, oxidation and nitration adducts are reported in Table 2. In subjects with ASD, protein content of AGEs - CML and CMA, and protein oxidation adducts - DT and NFK, were increased, with respect to children with TD. All increases remained significant after Bonferroni correction except for NFK. Plasma protein content of AGE, 3DG-H, was uniquely decreased in children with ASD, with respect to children with TD, remaining significant after Bonferroni correction.

**Table 2:** Glycation, oxidation and nitration adduct residue content of plasma protein of children with typical development or autism.

Protein modification marker	TD	ASD	*P* value	
FL (mmol/mol lys)	4.66 ± 2.50	4.34 ± 2.40	NS	
CML (mmol/mol lys)	0.123 ± 0.044	0.141 ± 0.054	<0.001**	
CEL (mmol/mol lys)	0.040 ± 0.025	0.040 ± 0.028	NS	
G-H1 (mmol/mol arg)	0.163 ± 0.120	0.169 ± 0.110	NS	
MG-H1 (mmol/mol arg)	0.887 ± 0.340	0.841 ± 0.320	NS	
3DG-H (mmol/mol arg)	0.232 (0.180–0.290)	0.175 (0.140 – 0.230)	<0.001***	
CMA (mmol/mol arg)	0.154 ± 0.084	0.188 ± 0.081	<0.001***	
GSP (mmol/mol lys)	0.238 ± 0.160	0.215 ± 0.130	NS	
MetSO (mmol/mol met)	7.55 (0.67–11.30)	7.12 (0.70–10.69)	NS	
DT (mmol/mol tyr)	0.017 (0.009–0.030)	0.026 (0.010–0.050)	<0.001***	
NFK (mmol/mol trp)	1.23 (0.83–1.74)	1.32 (0.91–2.21)	<0.05	
3-NT (mmol/mol tyr)	0.008 (0.006–0.012)	0.008 (0.005–0.011)	NS	

*NS* not significant.

Data are mean ± SD or median (lower—upper quartile); TD, *n* = 167, and ASD, *n* = 311. Significance (Students *t* test or Mann–Whitney U); ** and ***, *P* < 0.01 and *P* < 0.001 after Bonferroni correction of 12 applied.

Correlation analysis of plasma protein adduct levels with subject age are given in Table 3. Five of eight correlations of protein adduct levels with age in children with TD were negative with correlation coefficient r values ranging from –0.34 to –0.16. Eight of 10 correlations of protein adduct levels with age in children with ASD were negative with r values ranging from –0.35 to –0.13. Only CEL, G-H1 and 3DG-H correlated positively with subject age. Where correlations of a protein glycation or oxidation adduct with age were found for both children with ASD and TD, the direction of relationship (positive or negative) was the same for both groups. 3DG-H correlated positively with age only in subjects with TD and FL, NFK and 3-NT correlated negatively with age only in children with ASD.

**Table 3:** Correlation of glycation, oxidation and nitration adduct residue content of plasma protein with subject age of children with typical development or autism.

Protein modification marker	TD		ASD	
	r	*P* value	r	*P* value
FL			–0.21	<0.001**
CML	–0.28	<0.001**	–0.19	<0.001**
CEL	0.25	<0.001*	0.28	<0.001***
G-H1	0.17	<0.05	0.16	<0.01*
3DG-H	0.19	<0.05		
CMA	–0.26	<0.001**	–0.26	<0.001***
GSP	–0.34	<0.001***	–0.28	<0.001***
MetSO	–0.16	<0.05	–0.19	<0.01*
DT	–0.29	<0.001**	–0.28	<0.001***
NFK			–0.13	<0.05
3-NT			–0.35	<0.001***

Data are Spearman correlation coefficients for correlation of variable with subject age; TD, *n* = 167, and ASD, *n* = 311. Significance: *, ** and ***, *P* < 0.05, *P* < 0.01 and *P* < 0.001 after Bonferroni correction of 12 applied (there were no significant correlations with MG-H1).

We explored the correlation of severity of ASD with age and plasma protein glycation and oxidation markers with data from ADOS-2 assessment in the QBRI cohort. ADOS-2 score correlated negatively with age (r = −0.39, *P* < 0.001) and positively with plasma protein CEL (r = 0.20, *P* < 0.05) and MG-H1 (r = 0.20, *P* < 0.05).

### Validation and development of diagnostic algorithms for ASD

**Fig. 2:**
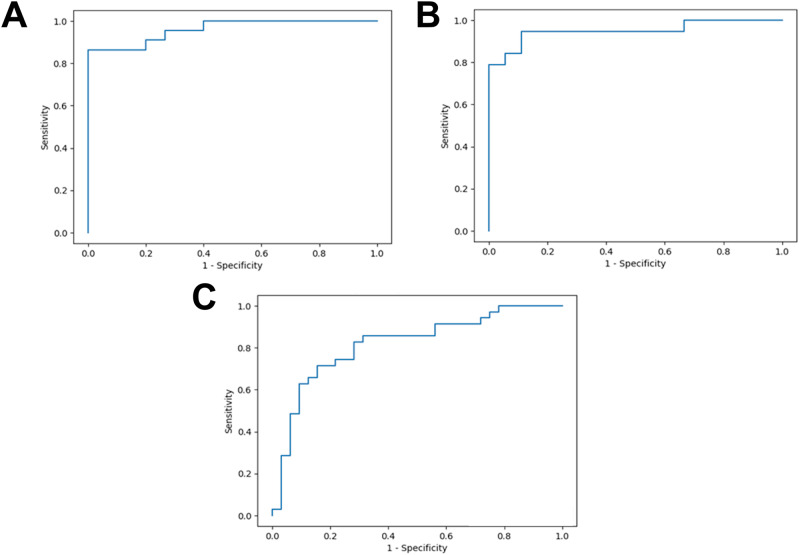
Receiver operating characteristic plots of diagnostic algorithms for detection of autism spectrum disorder by plasma protein glycation and oxidation adducts. **A** Classification of children with ASD or TD, 5 – 12 years old with features: age, gender CML, 3DG-H, CMA and DT (Algorithm 1; AUROC 0.89). **B** Classification of children with ASD or TD, 5 – 12 years old with features age, gender CML, 3DG-H, CMA, DT and AVE (Algorithm 2; AUROC 0.95). **C** Classification of children with ASD or TD, 1.5 – 12 years old with features: age, gender and all plasma protein glycation and oxidation markers (Algorithm 3; AUROC 0.82).

To validate the ASD diagnostic algorithm with plasma protein glycation and oxidation features, we initially used plasma protein modification analyte data of subjects 5–12 years old. Ensemble was the best-performing algorithm development method. The best classifier algorithm had the following features: age, gender and CML, CMA, DT and 3DG-H. Classification performance was: accuracy 83%, sensitivity 94%, specificity 67% and area-under-the-curve of receiver operating characteristic plot (AUROC) 0.87 (Table 4 and Fig. 2a). We explored if addition of other plasma protein glycation and oxidation features added to the algorithm improved the classification performance. The best outcome was with addition of an average of all plasma protein glycation and oxidation features, AVE, which increased accuracy and specificity. Classification performance: accuracy 84%, sensitivity 91%, specificity 74%, and AUROC 0.89 (Table 4 and Fig. 2b).

**Table 4:** Diagnostic algorithms developed for autistic spectrum disorder from plasma protein glycation and oxidation adducts.

Algorithm no	1	2	3
Subject age range (years)	5–12	5–12	1.5–12
Features	Age, gender CML, 3DG-H, CMA & DT	Age, gender CML, 3DG-H, CMA, DT and AVE	Age, gender, FL, CML, CEL, CMA, G-H1, MG-H1, 3DG-H, GSP, DT, NFK and 3-NT
Accuracy (%)	83 (79–89)	84 (79–89)	74 (70–79)
Sensitivity (%)	94 (90–98)	91 (82–99)	75 (63–87)
Specificity (%)	67 (57–76)	74 (68–80)	74 (58–90)
AUROC	0.87 (0.84–0.90)	0.89 (0.82–0.96)	0.79 (0.74–0.84)
Positive likelihood ratio, LR+	3.02 (2.23–3.81)	3.55 (2.63–4.47)	3.42 (2.01–4.82)
Negative likelihood ratio, LR-	0.09 (0.04–0.15)	0.13 (0.01–0.24)	0.33 (0.20–0.46)
Positive predictive value (%)	81 (72–90)	87 (82–91)	74 (62–86)
Negative predictive value (%)	86 (76–96)	81 (62–99)	77 (69–85)
F-score	0.87 (0.82–0.91)	0.88 (0.84–0.92)	0.73 (0.68–0.79)

Algorithm outcomes for fivefold cross-validation (10 randomized repeat trials for robustness) using Ensemble. Data are mean (95% CI).

We also explored the application of algorithms based on plasma protein glycation and oxidation features to subjects over the wider age range of 1.5–12 years. The classification performance declined yet achieved upper limits of CIs for specificity and sensitivity exceeding the APA Work Group classification quality threshold of 80%. The best classification achieved was with features: age, gender and all plasma protein glycation and oxidation features measured (FL, CML, CEL, CMA, G-H1, MG-H1, 3DG-H, GSP, MetSO, DT, NFK and 3-NT). Classifier performance was: accuracy 74%, sensitivity 75%, specificity 74% and AUROC 0.79 (Table 4 and Fig. 2c).

## Discussion

The primary objective of this study was to validate the outcome of our discovery biomarker study of ASD diagnosis via replication in additional cohorts of age range 5–12 years old [17]. The remarkable outcome from this validation study is that training and testing algorithms based on the same 4 biomarkers, CML, CMA, DT and 3DG-H, of plasma protein analysed in subjects recruited at 3 centres and two countries different from the discovery study produced an algorithm of similar classification performance. With accuracy of 83%, LR + 3.0 and LR- 0.09, the diagnostic test provides moderate evidence for presence of ASD and strong, often convincing evidence of absence of ASD (Table 4). It is therefore particularly good for identifying absence of ASD and thereby allowing child development experts to stratify attention to children that likely have ASD for further follow-up interview and observation. Including additional protein glycation, oxidation and nitration adducts improved the specificity such that the 95%CI met the APA Work Group classification quality threshold of 80%. The reason for the improvement in specficity is unclear but additional input on changes in protein glycation and oxidation status may reflect the association of ASD with increased fasting plasma glucose (FPG) and insulin resistance [29] and susceptibily to activaton of the unfolded protein response (UPR) [30, 31] – see below. The successful validation indicates that the combination of protein glycation and oxidation biomarkers may find diagnostic application in early, laboratory-based screening for potential cases of ASD.

Our diagnostic test is generally applicable with a zero no response rate and 100% test validity rate, requiring a small aliquot of venous blood. In the current study, subject age and gender were also features contributing to the ASD and TD classification accuracy of the diagnostic alogorithm – although not in the discovery study [17]. We attribute this to the increased cohort size and related statistical power, and increased dispersion of subject age across the inclusion age range criteria.

Our approach is unique in focussing on biomarkers based on spontaneous modifications of plasma protein by glycation and oxidation as biomarkers for the diagnosis of ASD (Table 2). Increased CML and CMA likely reflects increased lipid peroxidation [32] – in agreement with earlier studies of increased plasma malondialdehyde and increased urinary isoprostanes in subjects with ASD [33–36]. Increased DT likely reflects increased activity of dual oxidase which catalyses the formation of DT and has been linked to host immunity and neuronal plasticity [37, 38]. Decreased 3DG-H may reflect increased activation of the UPR and increased clearance of 3DG-H-modified proteins. Proteins modified by reactive dicarbonyl metabolites such as 3-deoxyglucosone are misfolded and activate the UPR [30, 31]. Notably, a study of postmortem brain tissue of subjects with ASD revealed increased expression of proteins of the UPR [39]. Finally, decreased 3DG-H modification of albumin may be an indirect marker of CNS activation of the UPR in the brain of subjects with ASD. Mechanisms of formation of these plasma protein adducts may be prospective targets for therapeutic intervention in ASD. If so, our test may find future application in therapeutic monitoring.

Plasma protein modification anlaytes used herein have advantages over other biomarkers. Firstly, by measuring modifications on mainly albumin in plasma, in exchange with albumin of the CSF, we have biomarkers potentially reporting on changes in metabolites and protein modification status within the brain where neuronal dysfunction in ASD originates [1]. Secondly, plasma protein glycation and oxidation adducts provide a cumulative report on metabolic dysfunction related to the processes of their formation over 3 – 4 weeks prior to sampling; *cf*. the use of glycated albumin to assess glycemic status [19].

A secondary objective of the current study was to explore the application of plasma protein glycation and oxidation biomarkers to the classification of children with ASD or TD of a lower minimum inclusion age, 1.5 years, than in the discovery study [17]. Including this lower limit of subject age produced a decreased classification performance of algorithms – although inclusion of additional protein glycation and oxidation markers gave upper limits of 95% CI exceeding 80% for both sensitivity and specificity. The accuracy and AUROC of this classification was decreased compared to algorithms for the 5–12 year age group (*cf*. algorithm-3 and algorithms-1 and -2). This was linked to a lower sensitivity of 75% for algorithm-3. The reason for this is not clear but it may be linked to the differences in the correlation of protein glycation, oxidation and nitration adduct features with age between subjects with TD and autism (Table 3) leading to impaired classification for subjects of less than 5 years of age. From correlation analysis with age, most but not all plasma protein glycation, oxidation and nitration adducts correlated negatively with age. This may be linked to increased degradation and decreased half-life of albumin with age associated with increased plasma concentration of albumin in the age range studied [40]. For the 4 features of the mimimal algorithm, CML, CMA and DT correlated negatively with age in both children with ASD and TD whereas 3DG-H correlated positively with age in children with TD and did not correlate with age in children with ASD.

We explored the correlation of plasma protein glycation and oxidation biomarkrers with severity of ASD symptoms, as judged by the ADOS-2 score available in a subset of the cohort (Table 3). We found severity of ASD correlated negatively with subject age and postively with CEL and MG-H1. These AGEs are both formed from the reactive dicarbonyl glycating agent, methylglyoxal (MG). MG is formed as a byproduct of glycolysis and is increased with increased FPG and insulin resistance [41, 42]. This may suggest that severity of ASD is linked to increased exposure to MG. This is an interesting association deserving of further investigation.

Additional criteria of the APA Work Group on Neuroimaging Markers of Psychiatric Disorders for a diagnostic test were: good internal validity, external validity, and test-retest reliability and inter-rater reliability [6]. Our test meets these requirements. It is based on stable isotopic dilution analysis LC-MS/MS assay of small molecule protein glycation and oxidation adducts. This analytical technique is the gold standard reference technique for small molecule quantitation with high analytical sensitivity and specificity, robust calibration, and good reproducibility [43]. LC-MS/MS is often preferred for harmonization of analytic measurements between laboratories [44] and is regarded by the FDA as an appropriate analytical technology for Class 2 laboratory-based tests – such as diagnostic tests for ASD [45, 46].

The advance made in this study is a successful validation of the blood test for autism based on plasma protein glycation, oxidation and nitration adduct features in diagnostic algorithms for children 5–12 years of age (Table 4). This was achieved in a large multicenter clinical cohort study independent of the initial discovery phase study cohort. This provides evidence of high performance classification of children with autism and TD in support of regulatory approval and clinical use of the blood test. We have also extended the application of the blood test to children over a wider age range of 1.5–12 years. Our test is unique in using diagnostic biomarkers based on spontaneous post-translational modifications of proteins - glycation, oxidation and nitration adducts—with the potential for input into the test response of metabolic dysfunction in the central nervous system producing these modifications of proteins in the CSF exchanging with plasma.

When implemented clinically, diagnostic laboratories will be able to offer a validated blood test that may facilitate the screening and diagnosis of autism, aiding child development practitioners to make a referral within weeks. It would thereby help address long delays for autism diagnosis and enable experts in child psychology and development to focus on clinical care and follow-up of children with autism whilst being supported by a clinical chemistry blood test. Further developments envisaged in future studies are application of the blood test for risk prediction of autism progression to severe symptoms and application to therapeutic monitoring in alleviation of symptoms.

## Data Availability

Analytical data produced in this study may be obtained from the corresponding author.

## Code availability

We provide the code library in MATLAB^®^ and Python described in this work through Github.
